# Folic acid modified gelatine coated quantum dots as potential reagents for *in vitro *cancer diagnostics

**DOI:** 10.1186/1477-3155-9-50

**Published:** 2011-11-10

**Authors:** Valérie A Gérard, Ciaran M Maguire, Despina Bazou, Yurii K Gun'ko

**Affiliations:** 1 School of Chemistry, Trinity College Dublin, Dublin 2, Ireland; 2 School of Pharmacy and Pharmacology, Trinity College Dublin, Dublin 2, Ireland

**Keywords:** Quantum Dots, Folic acid, cancer, bio-imaging

## Abstract

**Background:**

Gelatine coating was previously shown to effectively reduce the cytotoxicity of CdTe Quantum Dots (QDs) which was a first step towards utilising them for biomedical applications. To be useful they also need to be target-specific which can be achieved by conjugating them with Folic Acid (FA).

**Results:**

The modification of QDs with FA *via *an original "one-pot" synthetic route was proved successful by a range of characterisation techniques including UV-visible absorption spectroscopy, Photoluminescence (PL) emission spectroscopy, fluorescence life-time measurements, Transmission Electron Microscopy (TEM) and Dynamic Light Scattering (DLS). The resulting nanocomposites were tested in Caco-2 cell cultures which over-express FA receptors. The presence of FA on the surface of QDs significantly improved the uptake by targeted cells.

**Conclusions:**

The modification with folic acid enabled to achieve a significant cellular uptake and cytotoxicity towards a selected cancer cell lines (Caco-2) of gelatine-coated TGA-CdTe quantum dots, which demonstrated good potential for *in vitro *cancer diagnostics.

## Background

Nanoparticles and especially quantum dots (QDs) have attracted much interest in recent years as potential diagnostics and drug delivery tools [1-3]. Thiol-stabilised CdTe semiconducting nanoparticles or quantum dots (QDs) present the particular advantage of being water-soluble and easy to functionalise [4,5]. In addition it has been shown that protective coatings such as gelatine may provide substantial improvement of their luminescence efficiency and biocompatibility [6,7]. They are therefore attractive for fluorescent bio-labelling, provided that they can be made specific to a target type of cell. In the present work, we have combined the improved biocompatibility provided by a gelatine coating with an increased uptake from cancerous cells over-expressing folic acid receptors. While the conjugation of folic acid (FA) to various nanoparticle types *via *a polymer spacer has been widely reported [8-13], here we describe a new, rapid, one-pot synthesis of folic acid-conjugated gelatine-coated TGA-capped CdTe QDs. The uptake of the resulting particles by cancer cells was assessed in Caco-2 cells which naturally over-express folate receptors (FR)[14].

For clarity purposes, gelatine-coated TGA-capped CdTe will be referred to as QD(A), gelatine-coated TGA-capped CdTe QDs with incorporated FA as QD(B) and gelatine-coated TGA-capped CdTe to which FA was conjugated *via *1-ethyl-3-(3-dimethylaminopropyl) carbodiimide **(**EDC) coupling as QD(C).

## Results and Discussion

### Synthesis and characterisation of folic acid-conjugated gelatine-coated CdTe QDs

**Figure 1:**
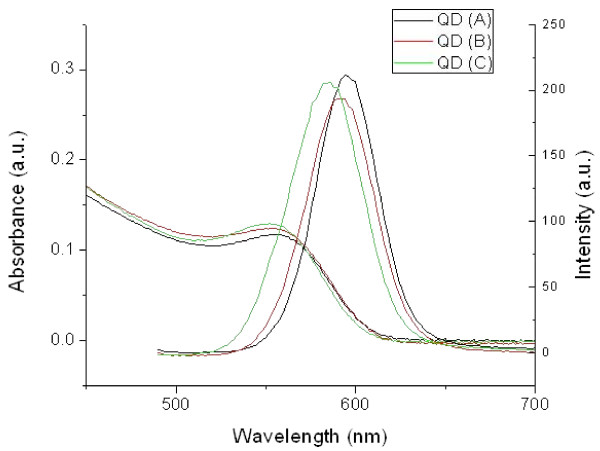
**UV-visible absorption and PL emission spectra of QD (A), (B) and (C)**.

Samples of QD(A), (B) and (C) were selected with similar spectroscopic properties: their maximum absorption (emission) wavelengths were respectively 556 (594), 554 (594) and 552 (586) nm, as shown on Figure 1. A quantum yield of 19%, 19% and 21% was recorded for QD (A), (B) and (C) respectively. The quantum efficiency was considered satisfactory for biological imaging.

**Figure 2:**
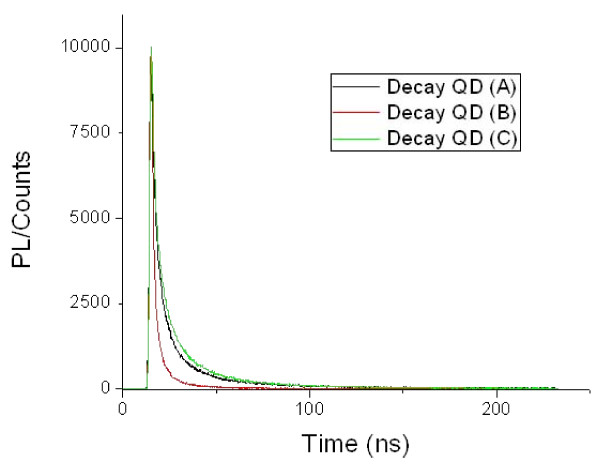
**Luminescence life time decay curves of QD (A), (B) and (C) at their maximum PL emission wavelength**.

Luminescence life time decay measurement provided further evidence of the surface modification. Figure 2 displays the luminescence lifetime decay curves. The shorter (T_1_) and longer (T_2_) lifetimes from the biexponential fit are presented in Table 1 along with their respective contributions (B_1 _and B_2_). QD (B) exhibited much shorter life times than QD(A) although they had the same quantum yield. T_2 _is associated with the surface state recombination of charge carriers. Therefore, a shorter T_2 _meant the surface defects and hence non-radiative pathways, had been modified although not eliminated since the luminescence efficiency had not increased. This was consistent with the presence of FA molecules in the gelatine layer. QD (C) showed again different life times from (A) and (B). It could thus been concluded that our synthesis had successfully produced three types of QDs with different surface modifications.

**Table 1:** Luminescence lifetime decay measurements.

Sample	T_1 _(ns)	T_2 _(ns)	B_1_	B_2_	CHISQ
QD (A)	3.29	14.77	30.95	69.05	1.200966
QD (B)	0.79	3.76	14.59	85.51	1.029932
QD (C)	1.82	9.28	12.18	87.82	1.116984

T_1_: shorter lifetime; T_2_: longer lifetime; B_1_: relative contribution of T_1_; B_2_: relative contribution of T_2_; CHISQ: Chi-squared

The three types of QDs were further characterized by Dynamic Light Scattering (DLS) and Zeta Potential measurements. Results are presented in Table 2.

**Table 2:** Size of QDs as measured by TEM and DLS, and their zeta potential.

Sample	Core diameter (nm)	Hydrodynamic diameter (DLS by number) (nm)	Polydispersity index (PDI)	Zeta potential (mV)	Standard deviation of Zeta potential
QD (A)	4.2 (+/- 0.7)	8.2	0.570	-19	1.7
QD (B)	4.8 (+/- 0.8)	27.7	0.312	-52	2.7
QD (C)	4.9 (+/- 0.9)	4.7	0.195	-20	4.1

**Figure 3:**
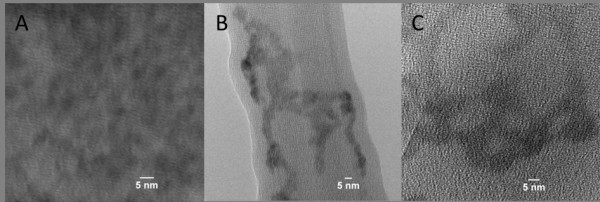
**TEM images of QD (A), (B) and (C) (left to right)**.

**Figure 4:**
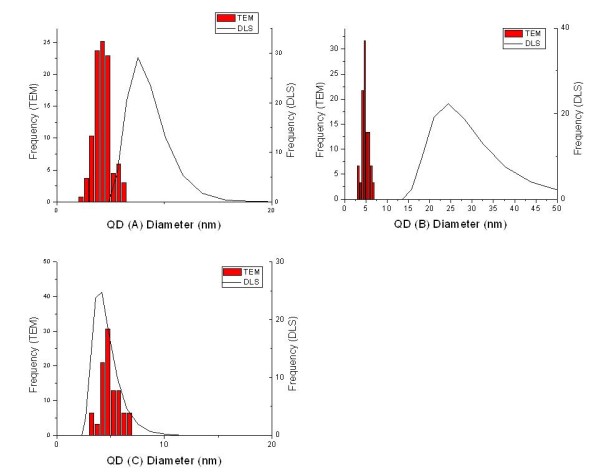
**Size distribution of QD (A), (B) and (C) as measured by TEM and DLS**.

The presence of organic material on the surface strongly influences DLS measurement as it affects the water shell that surrounds each particle as they move in solution. It does not however impact the core size of the particles measured on TEM images shown in Figure 3. This is why there are significant discrepancies between the core and hydrodynamic diameters as pictured on Figure 4. In the case of QD (A), the gelatine shell is responsible for the hydrodymic diameter being more than double the core diameter. QD (B) had very large hydrodynamic radius and zeta potential compared to the two other types. This accounted for effective incorporation of FA in the gelatine layer. Since the FA molecule is quite bulky it is expected that part of it should be sticking out of the gelatine shell, thus being potentially available for recoginition but also increasing the hydrodynamic radius of the particles. The presence of FA on the surface also lead to an increase in the surface charge owing to the two carboxylic groups per FA molecule. The better stabilisation implied by the high zeta potential was also reflected in the lower polydispersity index (PDI).

QD (C) was prepared by treating QD (A) with EDC in order to covalently bound FA to gelatine. One side effect of the treatment is the cross-linking of gelatine through intra- and inter-molecular reactions of carboxylic groups with amino groups of the protein [15,16]. This lead to reduced swellability of gelatine and hence a smaller hydrodynamic radius[15] as confirmed by the present results, as well as to less carboxylic groups available on the surface. This explains why the surface charge was rather low despite the presence of FA molecules.

### Biological testing of nanocomposites

**Figure 5:**
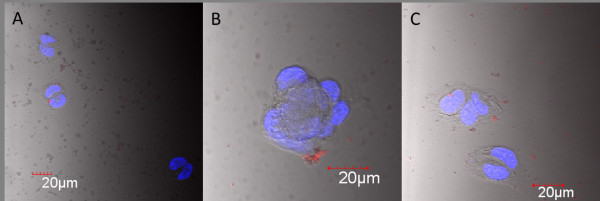
**Confocal microscope images of Caco-2 cells stained with DAPI (blue) and treated with QD(A), QD(B) and QD (C)**. (QDs are red).

The spontaneous cell uptake of QD(A), (B) and (C) was investigated and compared in Caco-2 (human colon adenocarcinoma) cells. Confocal microscopy images of treated cells are shown in Figure 5.

Caco-2 cells were previously reported to not efficiently take up a variety of nanoparticles[17]; however, since they are known to over-express folate receptors[14], the folic acid molecules present on the surface of particles were expected to significantly increase the uptake by these cells.

**Figure 6:**
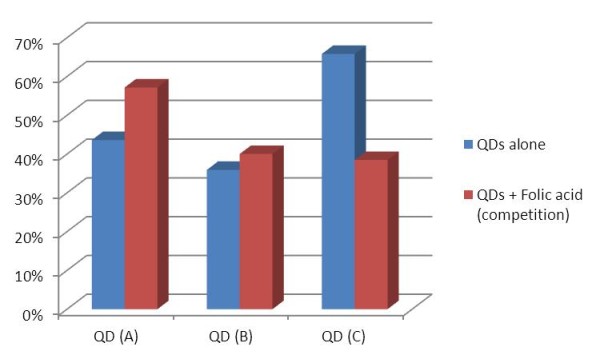
**Percentage of cells exhibiting internalised QDs in presence and absence of free FA**.

As shown in Figure 6, QD (A) and (B) were very similarly uptaken by the cells, with around 40% of cells exhibiting internalised QDs. Incorporated FA appeared to have no significant effect on particle uptake, which is understandable as the FA molecules would have random orientations and be partially trapped in gelatine, therefore the recognition site may not be available to bind to the receptors. On the other hand, QD (C) where FA molecules were covalently bound to the gelatine shell through their terminal amine, displayed a higher uptake of 66%.

To confirm that the increased upatke was related to FA, a competition assay was performed with free FA. In the case of QD (C) internalisation was reduced by free FA to the same level as QD (A) alone. As expected the free FA molecules could block the cellular receptors and QD (C) could only be internalised by unspecific endocytosis. On the other hand, the uptake of QD (A) was raised by the presence of free FA almost to the level of QD (C) alone. In this case, free FA could bind to gelatine thus dragging the particles into the cells. The uptake of QD (B) was not significantly altered by free FA because the surface was probably already saturated in randomly orientated FA molecules. Overall it could be reasonably concluded that the increase in uptake was directly linked to the presence of FA on the surface of the particles. QD (B) also proved to be of very little interest for biological applications.

Finally, preliminary cytotoxicity studies were conducted on QDs (A) and (C) in presence and absence of free FA using a Calcein AM viability assay. The results are shown in Table 3. Calcein AM is a fluorescent dye which is able to penetrate the cell membrane. Its fluorenscence is only released upon action of esterases in the cytoplasm. Since only viable cells produce active esterases, it can be used to assess cytotoxicity[18].

**Table 3:** Cytotoxicity of FA modified QDs towards Caco-2 cells.

Type	QD(A)	QD(A) +FA	QD (C)	QD(C) +FA
Cell death	23%	32%	59%	48%
Uptake	44%	57%	66%	38%

Thiol-stabilised aqueous CdTe QDs have been reported to be generally more toxic than ones produced through the organic route due to their lack of protective shell[19]. Adding a layer of gelatine however was found to reduce their cytotoxicity [6] which is believed to arise mainly from the release of cadmium ions[19]. Another critical aspect in QD toxicity is the size of the particles. In our study we used large, red-emitting QDs which have been reported to be less toxic than smaller ones, mostly because they are not able to penetrate as deep in the cell[20]. The cytotoxicity of our QDs appeared to be related to their uptake rate to a certain extent. FA-modified QDs however tend to be more cytotoxic than bare gelatinated QDs, which may be explained by their blocking of the FA receptors thus depriving the cells from this essential nutrient. This make them potential candidates for targeted cancer therapy, but more in-depth biological studies would be required in order to guarantee good enough specificity.

## Conclusions

In conclusion, all characterisation analyses that were carried out (UV-visible absorption spectroscopy, PL, DLS, zeta potential, fluorescence lifetime decay) pointed towards the effective modification of the gelatine-TGA CdTe QD surface with FA, using our approach. The most definite proof remains the competitive uptake of FA and QDs which demonstrated that variations were linked to the presence or absence of FA on the surface of particles. To some extent, the molecule can be incorporated to the gelatine shell; however the availability of FA for recognition was only obtained by covalent conjugation. We have thus developed a new potential assay for *in vitro *cancer diagnostic by identifying cells which highly express FR as it is the case for most carcinoma cells[21]. This is also a proof of concept for a new facile, efficient, one-pot synthesis of functionalised QDs which could be used to create combined diagnostics and therapeutic tools.

## Methods

### Materials

Al_2_Te_3 _was purchased from Cerac Inc. All other chemicals for synthesis were purchased from Sigma-Aldrich. All synthetic procedures and sample preparation were performed using degassed Millipore water. Caco-2 cells were purchased from the European Cell Culture Collection (ECCC).

### Synthesis of QD (A), (B) and (C)

QD (A), (B) and (C) were synthesised using a modification of the procedure previously reported by our group[7]. Briefly, the gelatine coated QDs were prepared by passing H_2_Te gas through an aqueous basic solution containing Cd(ClO_4_)_2_, thioglycolic acid (TGA) stabilizer. The resultant mixture was heated under reflux for 2 hours. The solution was then cooled to 80°C and divided into three flasks, A, B and C. Folic acid (0.01 moles, 0.28 g) was added directly to Flask B and the solution was stirred for 15 min. EDC (0.1 g) and DMAP (0.1 g) were added to flask C and the solution was stirred for 15 mins to activate the QDs for conjugation. Folic acid (0.01 moles, 0.28 g) was then added, and the mixture was allowed to react for 15 min, while stirring. From each of the crude solutions A, B and C, different fractions were precipitated out using 2-isopropanol and centrifuging (3000 rpm, 10 mins). Unreacted materials were removed by purification on a Sephadex column.

### Biological testing

Caco-2 cells were cultured in appropriate medium (500 mL Minimum Essential Medium (MEM) supplemented with 0.055 g of sodium pyruvate, 5 mL of a solution of penicillin (2 mM) and streptomycin (2 mM), 5 mL of 1 mM gentamicin and 100 mL of Fetal Bovine Serum (FBS)) at 37°C and in a 5% CO_2 _atmosphere. 80% confluent cell cultures were trypsinised and re-suspended in cell culture medium to a final concentration of 1.10^5 ^cells/mL and seeded on cover slips. After 24 h incubation allowing the cells to adhere to the substrate, half of the medium was removed from each dish and replaced by the same volume of serum-free medium. The cells were incubated for a further 4 h before the medium was aspirated out and replaced with 2 mL of QD suspension in Dubelcco's modified Phosphate Buffer Saline (DPBS) at a final concentration of 10^-7 ^mol/L. After four more hours, the QD containing solution was aspirated out of the dishes and the cells were washed three times with PBS. They were then fixed with 70% ethanol and mounted on slides using Vectashield mounting media containing 4',6-diamidino-2-phenylindole (DAPI). For FA competition experiments, FA at a final concentration of 10^-7 ^mol/L was added to the cell cultures along with QDs. Control cultures in DPBS without QDs, and with or without FA accordingly were also analysed.

### Cytotoxicity assay

Caco-2 cells were seeded as before and treated with QDs in the same conditions. After 4 h incubation, the QD containing solution was aspirated out of the dishes and the cells were washed three times with PBS. 50 μg of Calcein AM were dissolved in 50 μL of dimethyl sulfoxide (DMSO). The resulting 50 μL of solution were diluted in 10 mL of DPBS. 1 mL of dilute Calcein AM was added to each dish and incubated at room temperature for 30 min. The staining solution was aspirated out and the cell cultures were washed three times with PBS. Live cells, stained in green, were imaged using a confocal microscope, counted and compared to control cultures.

### Characterisation

A Shimadzu UV-1601 UV-Visible Spectrophotometer was used to measure QD absorption spectra. Scans were carried out in the 300-700 nm range. A Varian - Cary Eclipse Fluorescence Spectrophotometer was used to determine the photoluminescence (PL) emission spectra of QDs. The excitation wavelength was 480 nm and the emission was detected in the range 490-700 nm. The Quantum Yields (QY) were calculated from the PL spectra using Rhodamine 6 G as a reference. Hydrodynamic radii and zeta potential of nanoparticles were measured on a Malvern Zetasizer Nano Series V5.10. Five measurements were usually taken for each sample, each made of 10 to 20 accumulations as optimised by the machine. Fluorescence lifetime decays were measured using time-correlated single photon counting (TCSPC) on a Flurolog 3 Horiba Jovin Yvon, with samples excited at 480 nm and decays measured to 10000 counts. Biexponential fitting was used to generate the decay curves. A Jeol 2100 Transmission Electron Microscope (TEM) was used to image the CdTe QDs. Sizes of the nanoparticles were measured using ImageJ software. An Olympus FV1000 Point-Scanning Confocal Microscope was used to examine the cells after staining with QDs and counter-staining with DAPI or Calcein AM. Sequential acquisition was used to acquire the two colour images which were overlaid and analysed using the Olympus Fluoview version 7B software.

## Competing interests

The authors declare that they have no competing interests.

## Authors' contributions

VAG participated in the design of the study and in the QD characterization, carried out QD synthesis and characterization and biological testing and drafted the manuscript. CMM carried out QD synthesis and characterization. DB participated in conceiving the biological testing and interpreting the data. YKG conceived the study, participated in its design and coordination and helped in writing the manuscript. All authors read and approved the final manuscript.
